# Selection and techno-economic analysis of hybrid ground source heat pumps used in karst regions

**DOI:** 10.1177/0036850420921682

**Published:** 2020-05-18

**Authors:** Weisong Zhou, Peng Pei, Ruiyong Mao, Haibin Qian, Yanbing Hu, Jin Zhang

**Affiliations:** 1 College of Mines, Guizhou University, Guiyang, China; 2 School of Civil Engineering, Guizhou University, Guiyang, China; 3 Beijing Talent New Energy Technologies Ltd., Beijing, China; 4 Institute of Groundwater and Earth Sciences, Jinan University, Guangzhou, China

**Keywords:** Hybrid ground source heat pump, ground-coupled heat pump, groundwater heat pump, heat imbalance, karst region, economic analysis

## Abstract

In order to take advantage of different forms of heat pumps and to mitigate thermal imbalance underground caused by long-term operation of ground source heat pumps, hybrid ground source heat pump systems have received an increasing attention. In this research, based on the fact that abundant groundwater resources are commonly available in karst regions, a new strategy is introduced for selecting and determining hybrid ground source heat pump capacity. Five scenarios of hybrid ground source heat pump system coupling groundwater source heat pumps with other supplementary heat pumps are proposed in this article to provide appropriate options to eliminate heat buildup under different hydrogeologic conditions. Methodologies for sizing and selection are established. Then, a case study of techno-economic analysis was performed for a project in the karst region in South China. The results showed that these scenarios can effectively mitigate heat buildup, and under the hydrogeologic condition in the case study. Compared to the solo ground-coupled heat pump solution, the optimal solution (Solution 4 in this study) can reduce the annual costs by 16.10% and reduce the capital investment by 60%. Methodologies developed in this study are beneficial for selecting appropriate approaches to mitigate heat buildup and enhance competitiveness of ground source heat pumps.

## Introduction

Heat imbalance is one of the most important problems associated with the ground source heat pump (GSHP) and has a significant impact on system performance. In many cases, the heating and cooling loads can be very different due to varying climates and demands of users.^1^ For the ground-coupled heat pump (GCHP), the heat generated by compressors, fans, and pumps can be directly utilized in heating mode. While in the summer, the heat generated by the equipment adds extra heat rejected to earth, amplifying the heat imbalance underground. For example, the subtropical climate in some parts of South China is featured with a long, humid, and hot summer making the cooling load 1.5–3 times of heating load.

Various studies related to heat imbalance in subtropical regions have been conducted. Qian et al.^2^ studied the influence factors of underground thermal imbalance by selecting eight residences randomly located in the subtropical region. The authors pointed out that the key factors affecting the demands for cooling and heating are not only the operating temperature and starting temperature, but also the flexibility of the individual control by residents. Then, they conducted numerical simulations for different buildings, involving a series of operation modes, various terminal apparatus, and different load ratios. It was identified that the fan coil unit for both heating and cooling shows a 30%–40% lower imbalance ratio than fan coil units and radiant ceiling for either cooling or heating. In order to alleviate the thermal imbalance caused by GSHP air conditioner system, Li et al.^3^ proposed a system that coupled the GSHP with the water heater and identified through simulation work that the GSHP coupled with water heater can offset the extra heat injected to the ground. Zhao et al.^4^ used a transient simulation model to compare the GSHP with heat recovery, and they found that as the heat recovery rate increased, the thermal imbalance rate and soil temperature significantly decreased. The decline proves that the heat recovery geothermal heat pump (HP) system can effectively mitigate the thermal imbalance.

There are extensive karst areas in South China that belong to subtropical climate,^5,6^ and the abundant groundwater in these areas is an ideal heat resource for groundwater source heat pump (GWHP). However, so far very little attention has been paid to the effect of karst hydrogeological characteristics on GSHP projects situated in subtropical climates. Zeng et al.^7^ set up a test rig in Guilin city, China, to conduct experiments of GCHP system under different operation modes in a presentative karst environment. The results found that the temperature difference of two adjacent buried ground heat exchangers (GHEs) at a same depth was very different, and this was caused by the existence of groundwater flow in a karst structure where the GHE penetrated through. In another research of Zeng et al.,^8^ they concluded that the groundwater table may be influenced by joints and fractures of carbonate rock bodies, leading to fluctuation of groundwater table and saturation of soils, which in turn impacted heat exchange between GHEs and soils, as well as the performance of GSHP systems. Luo et al.^9^ investigated the thermo-economic performance of drilling cuttings grouted GHEs in karst area, and they concluded that the backfill material mixed with dolomite cuttings exhibited a lower thermal resistance. Nevertheless, some factors such as high drilling costs, immature construction techniques, and inappropriate drilling approaches^10^ block popularization of GSHPs in karst regions, and new methods need to be developed to enhance its competitiveness to other energy technologies.

HyGSHP (hybrid ground source heat pump) is supposed to mitigate the issue of ground thermal imbalance, as well as reduce the capital cost. In previous studies, the HyGSHP usually refers to a GSHP incorporated with a supplemental heat rejecter (cooling tower) to offset the excessive heat rejected to the ground. For example, since the annual cooling load exceeds the heating load in many commercial buildings in the United States, Kavanaugh^11^ proposed a design procedure to size the HyGSHP that involves a cooling tower. In order to balance the redundant heat injected to the ground, it is necessary to calculate the size of cooling tower and its operation time, and then correct the needed length of ground loop of GSHP.

**Figure 1.**
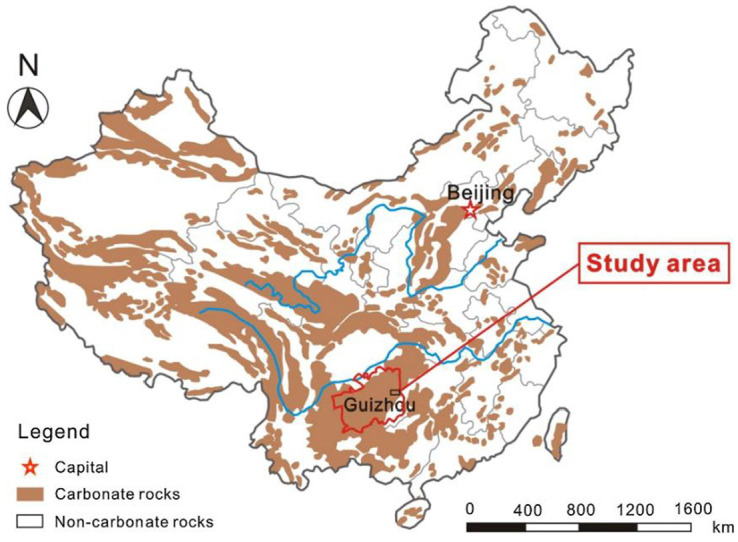
Distribution of the karst topography in China and location of the study area, modified from Luo et al.^9^

In this article, based on the hydrogeology characteristics in karst area, a new HyGSHP coupling GWHP and other types of HPs are proposed. A novel design procedure for this GWHP-based HyGSHP system and different coupling solutions are presented. On the same basis of an on-site building located in karst areas of South China of subtropical climate as shown in Figure 1, the technical and economic indicators of all solutions were compared with those of a solo GCHP system. It is identified that the scenarios and sizing methods proposed in this article could effectively balance the heat injection to and extraction from the ground, and reduce associated cost. It is expected that the research presented in this study will provide options to solve the heat imbalance problem of GSHP systems and promote a wider application of HP technology in karst regions.

## Description of HPs

**Figure 2.**
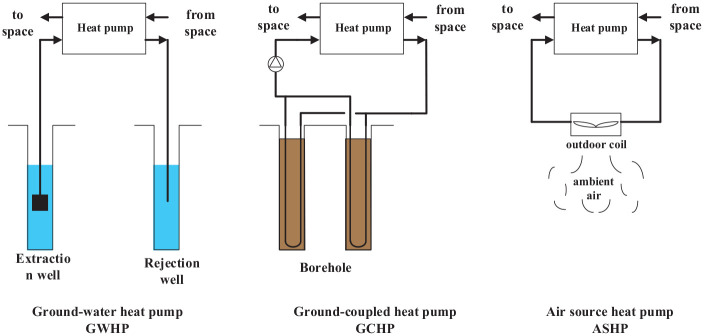
Schematics of different heat pumps.

HPs are applied to heating, ventilation, and air conditioning (HVAC) systems.^12^ The HP air conditioning (AC) system is more energy efficient and comfortable than traditional AC systems, and effectively reduces carbon emissions. The major difference between different types of HPs is their heat sources, which are depicted in Figure 2.

The HP involved in this article mainly includes GCHPs, GWHPs, and air source heat pumps (ASHPs). The GCHP has a high stability, but high capital investment and often causes ground heat buildup. The capital investment of GWHP is low, but its application is restricted by availability of water and environmental regulations.^13^ It is important to note that the term GWHPs in the traditional sense refers to these using groundwater from aquifers. However, in this article, GWHPs utilize groundwater in karst structures, such as caves and subterranean rivers. The ASHP system has a lower initial cost than the GCHP and GWHP systems, but the system performance is largely affected by the ambient air temperature which has a wide variation range.

## Methodologies

The basic concept of controlling ground heat balance in this article is converting the heating load of GCHP systems into heat rate extracted from the ground in the winter, which is then referred to calculate the amount of heat rejected by the GCHP system to the ground in the summer, and is used to calculate the cooling load of GCHP in the summer.

Based on the availability of water flow rate, five proposed HyGSHP scenarios are listed in Table 1, all of which are dominated by GWHPs using groundwater in karst structures.

**Table 1.** Proposed scenarios of HyGSHPs.

Scenario	Hybrid heat pump system/heat pump system	Requirement for water resource
1	Cooling mode: GWHPs Heating mode: GWHPs	Sufficient water available in summer and winter
2	Cooling mode: GWHPs + ASHPs Heating mode: GWHPs	Insufficient water in summer Sufficient water available in winter
3	Cooling mode: GWHPs + GCHPs Heating mode: GWHPs + GCHPs	Insufficient water in summer and winter
4	Cooling mode: GWHPs + GCHPs + ASHPs Heating mode: GWHPs + GCHPs	Insufficient water in summer and winter
5	Cooling mode: GWHPs + ASHPs Heating mode: GWHPs + boiler	Insufficient water in summer and winter

HyGSHP: hybrid ground source heat pump; GWHP: groundwater source heat pump; ASHP: air source heat pump; GCHP: ground-coupled heat pump.

**Figure 3.**
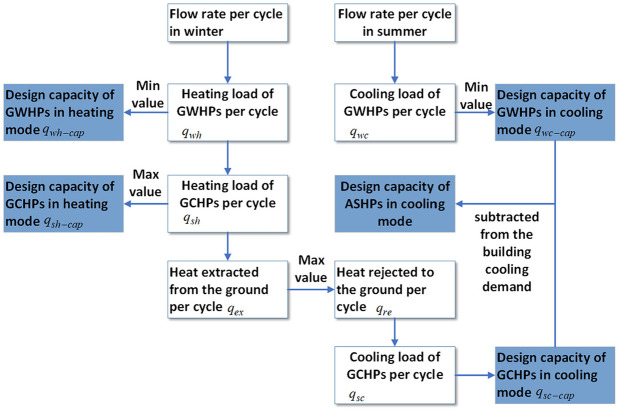
Calculation procedure.

First, the monthly heating and cooling periods were divided into four cycles according to variation pattern of flow rate of water sources. The installed capacity of GWHPs is determined by the minimum load among all cycles in winter, which is in turn determined by the lowest available water flow rate. Then, the installed capacity of GCHPs and ASHPs will be calculated subsequently. Such an approach is selected based on the consideration that the users’ load is fixed in the reference case, and coefficient of performance (COP) and operation cost of HP are directly affected by the load ratio. If a large capacity GWHP is selected, most of the time it will not be operated at full load, and consequently, its load ratio and COP would be decreased. Of course, such an issue can be offset by increasing the number of units, which, however, not only requires more footprints, but may cause more idle units most of the time. The method proposed in this article is to cope with the fluctuation of the groundwater. Therefore, the specific procedure is designed as follows:

The specific sizing process is presented as follows, which is also shown in Figure 3. Here, the heating demand $\left(q_{h}\right)$ refers to the total amount of the heat needed by the building. The heating load $\left(q_{\mathrm{wh}}\right)$ is the part of the heating demand that can be provided by the GWHP:

- The heating load of the GWHP $\left(q_{\mathrm{wh}}\right)$ was determined through the actual subterranean river flow rate in each winter cycle. Total building heating demand $\left(q_{h}\right)$ minus $q_{\mathrm{wh}}$ equals to the heating load of GCHPs in each cycle $\left(q_{\mathrm{sh}}\right)$ . The minimum value for $q_{\mathrm{wh}}$ was the heating design capacity of GWHPs $\left(q_{\mathrm{wh}-\mathrm{cap}}\right)$ , and the maximum value for $q_{\mathrm{sh}}$ was the heating design capacity of GCHPs $\left(q_{\mathrm{sh}-\mathrm{cap}}\right)$ .
- Then, the heat rate per winter cycle extracted from ground $\left(q_{\mathrm{ex}}\right)$ was obtained according to the value of $q_{\mathrm{sh}-\mathrm{cap}}$ . Based on the rule of making heat balance, $q_{\mathrm{ex}}$ was equal to the heat rate rejected to the ground per summer cycle $\left(q_{\mathrm{re}}\right)$ .
- The value of $q_{\mathrm{re}}$ was used to calculate the cooling load $\left(q_{\mathrm{sc}}\right)$ per cycle taken by GCHPs, which was also the cooling design capacity of GCHPs $\left(q_{\mathrm{sc}-\mathrm{cap}}\right)$ .
- The cooling load per cycle of GWHPs $\left(q_{\mathrm{wc}}\right)$ in summer was calculated in each cycle, and the cooling design capacity of GWHPs $\left(q_{\mathrm{wc}-\mathrm{cap}}\right)$ was the minimum value of the $q_{\mathrm{wc}}$ .

The calculation procedure is shown in Figure 3. If GWHPs and GCHPs still could not meet the building refrigeration demand in cooling mode, then ASHPs was utilized. The design cooling capacity of ASHPs was calculated by the building cooling demand minus $q_{\mathrm{sc}-\mathrm{cap}}$ and $q_{\mathrm{wc}-\mathrm{cap}}$ , respectively.

## Control equations

The required flow rate of GWHP units for heating is calculated as follows^13,14^

(1) $$G_{H}=\frac{Q_{H}}{c_{\mathrm{pw}}\left(t_{w1}-t_{w2}\right)}\left(\frac{\mathrm{CO}P_{h}-1}{\mathrm{CO}P_{h}}\right)$$

where $G_{H}$ (kg/s) is the required flow rate for heating, $t_{w1}$ (°C) is the entering water temperature or the temperature of groundwater, $t_{w2}$ (°C) is the leaving water temperature, $c_{\mathrm{pw}}$ (kJ/(kgK)) is the specific heat capacity at constant pressure of water, which is 4.19kJ/(kgK) , $Q_{H}$ (kW) is the heating load of buildings, and *COP_h_* is the coefficient of performance for heating of HP units.

The required flow rate of GWHP units for cooling is calculated as follows^13,14^

(2) $$G_{L}=\frac{Q_{L}}{c_{\mathrm{pw}}\left(t_{g2}-t_{g1}\right)}\left(\frac{\mathrm{CO}P_{c}+1}{\mathrm{CO}P_{c}}\right)$$

where $G_{L}$ (kg/s) is the required flow rate for cooling, $t_{g2}$ (°C) is the leaving water temperature, $t_{g1}$ (°C) is the entering water temperature, $Q_{L}$ (kW) is the cooling load of buildings, and *COP_c_* is the coefficient of performance for cooling of HP units.

The rate of heat extracted by GCHP system from the ground is calculated as follows^13^

(3) $$\frac{q_{\mathrm{ex}}}{q_{h}}=\frac{\left(\mathrm{CO}P_{\mathrm{hs}}-1\right)}{\mathrm{CO}P_{\mathrm{hs}}}$$

where $q_{\mathrm{ex}}$ (W) is the heat rate extracted by the HP system from the ground, $q_{h}$ (W) is the building design heating block load, and $\mathrm{CO}P_{\mathrm{hs}}$ is the coefficient of performance for heating of HP system.

The rate of heat rejected by GCHP system to the ground is calculated as follows^13^

(4) $$\frac{q_{\mathrm{re}}}{q_{c}}=\frac{\left(\mathrm{CO}P_{\mathrm{cs}}+3.412\right)}{\mathrm{CO}P_{\mathrm{cs}}}$$

where $q_{\mathrm{re}}$ (W) is the heat rate rejected by the HP system to the ground, $q_{c}$ (W) is the building cooling demand, and $\mathrm{CO}P_{\mathrm{cs}}$ is the coefficient of performance for cooling of HP system.

The resulting equation for GHE bore length can be estimated as follows^15^

(5) $$L=\frac{q_{\mathrm{cap}}}{a}$$

where *L* (m) is the required bore length, $q_{\mathrm{cap}}$ (W) is the installed capacity of GCHPs (the larger value between $q_{\mathrm{sc}-\mathrm{cap}}$ and $q_{\mathrm{sh}-\mathrm{cap}}$ ), and *a* (W/m) is the specific thermal exchange rate per unit length of borehole heat exchanger. According to the design manual of GCHP^15^ and communication with local GCHP operators, *a* is assumed as 60 in the study region.

The total annual cost, *AW* ($/year), is calculated by equation (6)^16^

(6) $$\mathrm{AW}=\frac{i{\left(1+i\right)}^{n}}{{\left(1+i\right)}^{n}-1}\times C_{0}+C$$

where *i* is the annual interest rate, which is assumed to be 8%, *n* is the service life of the system, $C_{0}$ (Chinese Yuan, CNY) is the initial capital investment, and *C* (CNY/year) is the cost of annual operation.

The thermal imbalance ratio, *IR*, is defined as follows^4^

(7) $$IR=\frac{q_{re}-q_{ex}}{Max\left(q_{re},q_{ex}\right)}\times 100\%$$

where $q_{\mathrm{re}}$ (W) indicates the total heat rejected to the ground, and $q_{\mathrm{ex}}$ (W) is the total heat extracted from the ground. IR is positive when the heat rejected to the ground is greater than the heat extracted, whereas IR is negative when the heat extracted from the ground is greater than the heat rejected.

## Case study

### Project background and assumptions

The examined project is a government complex located in the city of Tongren City of Guizhou Province, China—a karst region with 55% of the area covered by carbonate rocks. The total building area of this multipurpose complex is 80,000 m^2^, including some 24-h operation units, such as data center, urban security and facility monitoring, police duty, and emergency service. The building is designed with maximal cooling demand of 10,350 kW in the summer and maximal heating demand of 7500 kW in the winter, and its equivalent full-load hours are 13 h per day.^17^ The groundwater temperature is 16°C–20°C. The annual temperature of Tongren City varies from 13.5°C to 17.6°C, and the calculated wet bulb temperature, dry bulb temperature, and dry bulb temperature with ventilation are 26.7°C, 35.3°C, and 32.2°C, respectively. In winter, the calculated dry bulb temperature is −0.5°C and the calculated outdoor relative humidity is 76%. The performance of ASHP would be constrained by the climate in winter (frosting), so the ASHP is not considered for heating in winter. The cooling period was assumed from June to August, and the heating period was assumed from December to February next year. The units run 13 h a day. Two subterranean rivers nearby were assumed as the potential water source for GWHPs. According to the pattern of the flow rate of subterranean rivers, the heating (winter) and cooling (summer) periods were divided into 13 cycles (7 days per cycle), respectively. All needed parameters used for economic analysis are shown in Table 2.

**Table 2.** Parameters used for economic analysis.

Parameter	Assumed value	Unit	References
Drilling cost	100	CNY/m	Luo et al.^9^
Thermal exchange ratefor the borehole heat exchanger per meter	60	W/m	Ma and Lv^15^
Installation cost	15% of equipment cost	CNY	Chen^18^
Natural gas price	4.50	CNY/N m^3^	Lei et al.^19^
Electricity price	0.60	CNY/(kWh)	Guizhou Provincial Developmentand Reform Commission^20^
*COP_h_* of GCHP unit	5.00	–	ASHRAE^21^
*COP_h_* of GCHP system	3.70	–	ASHRAE^21^
*COP_c_* of GCHP unit	5.50	–	ASHRAE^21^
*COP_c_* of GCHP system	4.10	–	ASHRAE^21^
*COP_h_* of GWHP unit	4.50–4.80	–	ASHRAE^21^
*COP_c_* of GWHP unit	1.55–1.64	–	ASHRAE^21^
Annual interest rate	8%	–	–
Service life	20	Year	–

GCHP: ground-coupled heat pump; GWHP: groundwater source heat pump.

### Case analysis based on two subterranean rivers

According to Li and Tang,^22^ the change of subterranean river water flow rate slightly lags behind the precipitation. The flow rate is generally stable except in the raining season, and it is a feasible source of heating and cooling for the GWHP system.

The flow rate of River 1 (Changbao subterranean river)^22^ reached to a maximum value of 283 L/s in second cycle of summer and to a minimum value of 125 L/s in the last few cycles of August. The average flow rate in the summer was 179 L/s, with a variation range of 226%. The average flow rate in the winter was 139 L/s, with a variation range of 143%.

**Figure 4.**
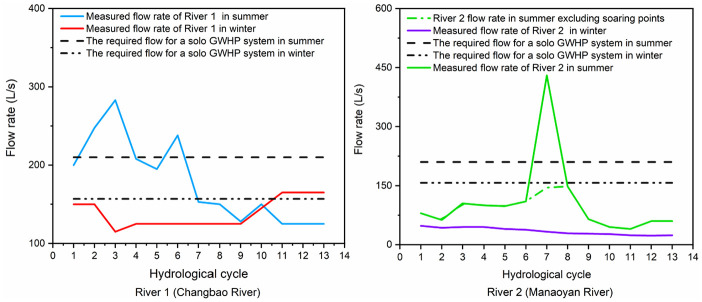
Flow rate variation of Changbao subterranean river and Manaoyan subterranean river.

While the flow rate of River 2 (Manaoyan subterranean river)^22^ was modified to remove several soaring points due to precipitation. The average flow rate in the summer and winter was 86.00 and 34.4 L/s, respectively, and they are lower than that of River 1. The variation range of flow rate was 370% in the summer and 210% in the winter. Figure 4 depicts their volumetric flow rate variation in cooling and heating periods.

It should be noted that the measured flow rate of the subterranean river in the report^21^ was not able to satisfy the solo GWHP for the project. But to show more solutions and compare their economic benefits, the solution of solo GWHP is presented in the following discussion.

#### System capacity calculation

For River 1, in Scenario 1, if the building cooling and heating loads were supposed to be fully supported by the GWHP system, the flow rate should be at least 210 L/s in the summer and 157 L/s in the winter, which cannot be satisfied by the subterranean river as shown in Figure 4, so Scenario 1 was excluded. Scenario 2 was also rejected since the flow rate in the winter cannot support the GWHP system for the full heating load. In Scenario 3, GWHPs needed to share a certain cooling load (9304 kW); so during the cooling period, the share of GCHP was reduced and heat rejection to and extraction from underground by the GCHP was balanced. Correspondingly, a flow rate of 262 L/s was required, and the subterranean river flow rate cannot satisfy the requirement for more than half of the cycles, hence the elimination of Scenario 3.

As for Scenario 4, following the calculation approach in Figure 3, the maximum value of $q_{\mathrm{sh}}$ was 2798 kW and the maximum heat extraction rate from the ground was 2041 kW, which was also the maximum heat rejection rate, then it determined the cooling load supported by GCHPs must be 1641 kW. According to the flow rate, the remaining cooling load supported by GWHPs and ASHPs could be determined.

In Scenario 5, the load of GWHP was calculated by the flow rate. A natural gas boiler was used as a supplementary in winter and ASHP was used in the summer. Thus, this scenario was feasible regardless of the water flow rate.

For River 2, since the flow rate of River 2 was much lower than River 1, Scenarios 1–3 were also rejected due to insufficient flow rate. The capacities of units in Scenarios 4 and 5 can be determined through the specific sizing process in section “Methodologies.”

On the basis of discussions above, the selected equipment and related parameters are presented in Tables 3 and 4.

**Table 3.** The selected equipment with parameters and cost for Scenario 4.

Equipment	GCHP	GWHP	ASHP
Quantity	2	2	44
Unit price (Thousand CNY)	535	1170	80
Total price (Thousand CNY)	1070	2340	3520
Rated power/unit (kW)			
Cooling	229	460	44
Heating	341	744	–

GCHP: ground-coupled heat pump; GWHP: groundwater source heat pump; ASHP: air source heat pump.

**Table 4.** The selected equipment with parameters and cost for Scenario 5.

Equipment	GWHP	ASHP	Boiler
Quantity	2	52	1
Unit price (Thousand CNY)	806.3	80	271.7
Total price (Thousand CNY)	1612.6	4160	271.7
Rated power/unit (kW)			
Cooling	317	44	–
Heating	503	–	18.5

GWHP: groundwater source heat pump; ASHP: air source heat pump.

According to the calculation results and equipment information provided by manufacturers (Tables 3 and 4), the design capacities and installed capacities of the two selected scenarios of two subterranean rivers above were determined and shown in Tables 5 and 6.

**Table 5.** The installed capacity of all units in all scenarios under the River 1 flow rate data.

Scenario	GCHP		GWHP		ASHP	Gas boiler	Total capacity	
	Heating	Cooling	Heating	Cooling	Cooling	Heating	Heating	Cooling
4								
Installed capacity (kW)	3392	3524	4992	4624	5200	–	8384	13,348
Design capacity (kW)	2798	1641	4703	3601	5108	–	7501	10,350
5								
Installed capacity (kW)	–	–	4992	4624	6760	2800	7792	11,384
Design capacity (kW)	–	–	4703	3601	6749	2798	7501	10,350

GCHP: ground-coupled heat pump; GWHP: groundwater source heat pump; ASHP: air source heat pump.

**Table 6.** The capacity of all units in all scenarios under the River 2 flow rate data.

Scenario	GCHP		GWHP		ASHP	Gas boiler	Total capacity	
	Heating	Cooling	Heating	Cooling	Cooling	Heating	Heating	Cooling
4								
Installed capacity (kW)	7023	6735	1458	1345	6110	–	8481	14,190
Design capacity (kW)	6409	3760	1091	1266	6022	–	7500	11,048
5								
Installed capacity (kW)	–	–	1458	1345	9100	7000	8458	10,445
Design capacity (kW)	–	–	1091	1266	9084	6409	7500	10,350

GCHP: ground-coupled heat pump; GWHP: groundwater source heat pump; ASHP: air source heat pump.

#### System cost analysis for case of two subterranean rivers

**Figure 5.**
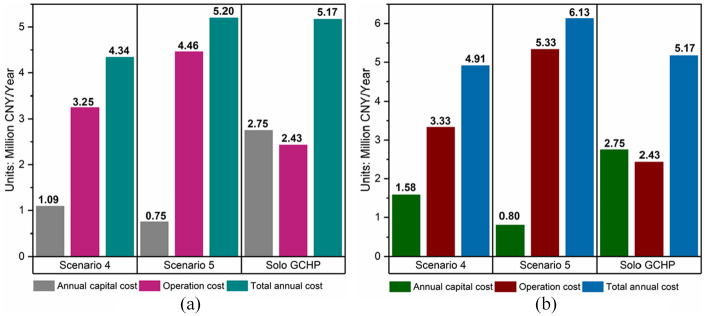
The annual cost of Scenarios 4, 5, and the GCHP system based on the flow rate of two subterranean rivers: (a) River 1 (Changbao river) and (b) River 2 (Manaoyan river).

According to Tables 2–6, the annual capital, operation cost, and total annual cost of two subterranean rivers were compared in Figure 5. For comparison, the cost of a solo GCHP system was also calculated as a baseline.

## Discussion

### Capacity of systems

As can be seen from Tables 5 and 6, in order to ensure ground thermal balance, the design capacity of GCHPs in heating and cooling modes in Scenario 4 differed significantly. Comparing the calculation result for cases of Rivers 1 and 2, it can be concluded that, when the available flow rate was higher, more load could be shared by GWHPs and less shared by GCHPs. Therefore, the gap of heating and cooling modes for GCHPs is in inverse proportion to the available water flow rate. In practical operation, a large gap between installed capacity of a unit and its actual load would result in waste of power and low energy efficiency. To avoid such a situation, it is recommended to install smaller units, which will lead to purchase of one more unit, and subsequently increase in capital investments, but the benefit is that the operation cost will drop and higher efficiency.

### Cost comparison

Costs in the case of River 2 were higher than those in the case of River 1. This is because of the fact that the water flow rate of River 2 was lower and more GHEs were needed to meet the total demand, and the cost of the buried pipe drilling was the major component of the capital investment of the GCHP.

In terms of capital investment, both Scenarios 4 and 5 offered a lower capital investment than a solo GCHP system. Especially, the capital investment of the Scenario 5 was significantly lower than others.

In terms of operation costs, boilers were used in the winter in Scenario 5, resulting in a higher operating cost than other options due to high natural gas price. The operation costs of Scenario 4 in both cases of Rivers 1 and 2 were still higher than those of a solo GCHP system by 25.33% and 27.06%, respectively. This is mainly due to the lower energy efficiency ratio (EER) and higher operation cost of ASHP used in Scenario 4.

In terms of total annual costs, the total annual cost of Scenario 4 was lower than that of a solo GCHP system (16.10% lower in the case of River 1 and 5.13% lower in the case of River 2). However, the total annual cost of Scenario 5 was higher than that of a solo GCHP system (0.56% higher in the case of River 1 and 5.13% higher in the case of River 2). This indicates that Scenario 4 is more economical regardless of available the flow rate, and Scenario 5 might not be a good option when the available water flow rate is low.

### Compare with solo GCHPs

According to equations (3), (4), and (7), it can be calculated that the solo GCHP system had a heat rejection rate of 12,874.39 kW in the summer, a heat extraction rate of 5472.97 kW in the winter, and the thermal imbalance ratio reached to 57.50%. To compare, Scenario 4 proposed in this article can limit the imbalance rate to nearly 0, and at the same time reduce the cost, while the Scenario 5 does not employ the GCHPs and there is no thermal imbalance problem, although the cost is relatively high.

## Conclusion

- In karst regions, the groundwater resources in karst structures are abundant, and it is possible to eliminate the problem of heat buildup using GWHPs and different HyGSHPs instead of solo GCHPs. Five scenarios based on GWHPs were proposed in this article, and the size selection and calculation approach was established.
- Through case analysis of techno-economics, Scenario 4 (summer: GWHPs + GCHPs + ASHPs, winter: GWHPs + GCHPs) is more cost-effective than the solo GCHP system. To specify, compared to the solo GCHP system, when the subterranean river flow rate in the case study was high (the average daily flow rate was 173.00 L/s in summer and the winter reached 139.00 L/s in River 1), the cost was reduced by 16.10%; while in the case of a river with a low flow rate (the average daily flow rate was 86.00 L/s in summer and the winter reached 34.40 L/s in River 2), the cost was reduced by 5.13%.
- When the flow is insufficient (refer to River 2 in the case study), it is not recommended to use the Scenario 5 (summer: GWHPs + ASHPs, winter: GWHPs + gas boilers), although the capital investment is low, but its operation cost would be boosted due to the use of boilers and ASHPs.
- In practical operation of GCHPs, heats generated by facilities such as pumps and compressors can make up for a portion of the heat used in heating mode, but these heats have to be rejected to ground together with the heat removed from the building in cooling mode. This amplifies the gap between the design cooling and heating capacities of the units, and also results in two consequences: (a) more units have to be installed, increasing the capital investment, and (b) the load factor of the units in cooling mode is lower, resulting lower energy efficiency.^23^ These questions should be considered in future work.

To conclude, the approach of size selection and calculation established in this study is useful for operators in determining the appropriate HyGSHPs when there is available water resource to utilize. The scenarios proposed in this article were able to balance the heat duty into the earth to effectively eliminate the heat buildup issue, and offered a lower cost than the solo GWHP system, enhancing its competitiveness of GSHPs on the market.
